# Cathodoluminescence imaging of cellular structures labeled with luminescent iridium or rhenium complexes at cryogenic temperatures

**DOI:** 10.1038/s41598-022-17723-w

**Published:** 2022-08-04

**Authors:** Marie Vancová, Radim Skoupý, Eva Ďurinová, Tomáš Bílý, Jana Nebesářová, Vladislav Krzyžánek, Aleš Kolouch, Petr Horodyský

**Affiliations:** 1 Institute of Parasitology, Biology Centre CAS, České Budějovice, 37005 Czech Republic; 2 Faculty of Science, University of South Bohemia, České Budějovice, 37005 Czech Republic; 3 Institute of Scientific Instruments CAS, Brno, 612 000 Czech Republic; 4 Faculty of Science, Charles University in Prague, Prague, 128 00 Czech Republic; 5 CRYTUR, Spol. S R.O., Turnov, 511 01 Czech Republic

**Keywords:** Biological techniques, Nanoscience and technology

## Abstract

We report for the first time the use of two live-cell imaging agents from the group of luminescent transition metal complexes (IRAZOLVE-MITO and REZOLVE-ER) as cathodoluminescent probes. This first experimental demonstration shows the application of both probes for the identification of cellular structures at the nanoscale and near the native state directly in the cryo-scanning electron microscope. This approach can potentially be applied to correlative and multimodal approaches and used to target specific regions within vitrified samples at low electron beam energies.

## Introduction

Electron microscopy reveals cellular structures in incredible detail; however, the specific localization of nanoscale structures in its native, cryo-fixed environment is still in its infancy. The current approaches for localizing the structures at room temperature include immuno- and affinity-gold labeling, and less often, chemical labeling^1^. Another possibility is incorporation of bioorthogonal labels, genetically expressed tags or fluorescent probes, whose detections are correlated to the ultrastructure in the same cell (correlative light and electron microscopy) with super-resolution microscopy and cryo-fluorescence microscopy^2–5^. These imaging technologies, which have advanced rapidly in recent years, provide significant progress in understanding how molecules function in the structural context.

Here, we explore the use of IRAZOLVE-MITO and REZOLVE-ER probes for cathodoluminescence (CL) imaging. CL is the emission of photons from a material in response to excitation by accelerated electrons. Detection of CL emission from biological specimens^6,7^, organic fluorophores^8–10^, proteins, and semiconductor quantum dots^11^ is extremely challenging due to low intensity optical signals that further bleach rapidly under the beam of accelerated electrons due to the structural damage of the biological material. In contrast, luminescent inorganic nanocrystals (e.g., nanodiamonds or rare-earth element-doped nanocrystals) have gained increasing importance as bright and stable CL probes with narrow emission spectra^12–18^. Rare earth element-doped nanocrystals can be potentially useful for correlative (multicolor) CL electron microscopy. Specifically, YVO_4_:Bi^3+^, Eu^3+^, and Y_2_O_3_:Tb^3^ nanocrystals were simultaneously detected with CL and BSE at low electron acceleration voltages (≤ 2 kV) within human vascular endothelial cells in epoxy resin blocks using Focused Ion Beam Scanning Electron Microscopy (FIB-SEM)^19^. FIB-SEM was used to detect LaF_3_:Tb^3+^ particles within endocytic compartments of human cells embedded in epoxy resin^12^. The nanocrystals (e.g., Y_2_O_3_∶Tb^3+^, Y_2_O_3_∶Eu^3+^, LaF_3_:Tb^3+^), which have around 10 nm in diameter, showed a remarkable resistance of the CL signal against electron beam exposure even at a high acceleration voltage (80 keV, 2 nA, 100 ms/pixel). They retained the CL intensity of more than 97% compared to the initial intensity for 1 min^20^.

The IRAZOLVE-MITO and REZOLVE-ER probes were designed for time-lapse imaging^21,22^. REZOLVE-ER, chemically *fac*-[Re(CO)_3_(1,10-phenanthroline)(4-pyridyltetrazolate)], specifically localizes to the nuclear membrane and endoplasmic reticulum and also allows the detection of exocytotic events at the plasma membrane^21^. IRAZOLVE-MITO (iridium complexed with cyclometalated 2-phenylpyridine and the 5-(5-(4-cyanophen-1-yl)pyrid-2-yl)tetrazolate ligand) has a high specificity for mitochondria in live cells, and its emission spectrum is in the range of 505–625 nm^22^. Both probes rapidly penetrate the cell membrane, have low cytotoxicity, are resistant to photobleaching, and in contrast to organic fluorophores, have long excited state lifetimes ranging between hundreds of nanoseconds to milliseconds^23^. The extended excited state lifetimes stem from the structural arrangement of the probes and excited triplet multiplicity nature of Ir and Re^21,22^. Transition metal complexes enable access to new electron states due to the presence of d-block metal centres. This gives rise to their unique photophysical and photochemical properties (high resistance to photobleaching, extended excited state lifetime, and low cytotoxicity over long exposure time), making these probes valuable for long-term live-cell imaging^23–25^.

## Results and discussion

**Figure 1:**
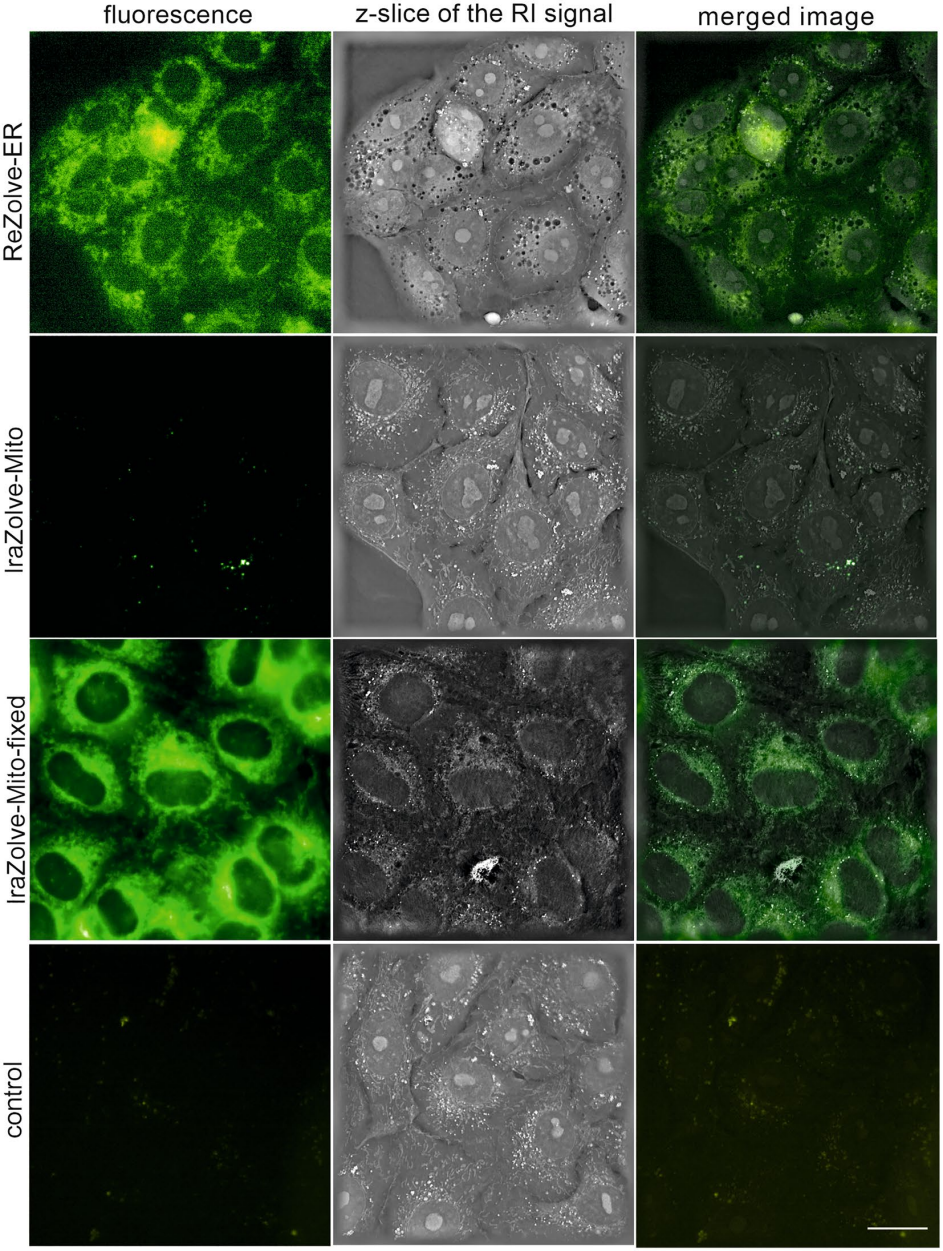
REZOLVE-ER and IRAZOLVE-MITO probes were used for imaging of endoplasmic reticulum and mitochondria of Vero cells, respectively. Holographic microscope 3D Cell Explorer (Nanolive). Bar 20 µm. RI (the refractive index).

We employed at first holographic microscopy in combination with fluorescence to visualize in vivo REZOLVE-ER and IRAZOLVE-MITO staining patterns in Vero cells. The dyes were used at concentrations 50 µM and 20 µM, respectively, and incubated for either 30 min or overnight (see Supporting Information). REZOLVE-ER staining was observed on the reticular network around the nucleus, whereas IRAZOLVE-MITO accumulated within vesicle-like structures, probably lysosomes. However, in cells that were pre-fixed before IRAZOLVE-MITO application, the staining pattern changed and the granular and filamentous structures extending throughout the cells were labeled (Fig. 1). A similar observation was seen in Vero cells stained with a mitochondrial stain MITOBLUE (fluorescent bisbenzamidine derivative)^26^.

**Figure 2:**
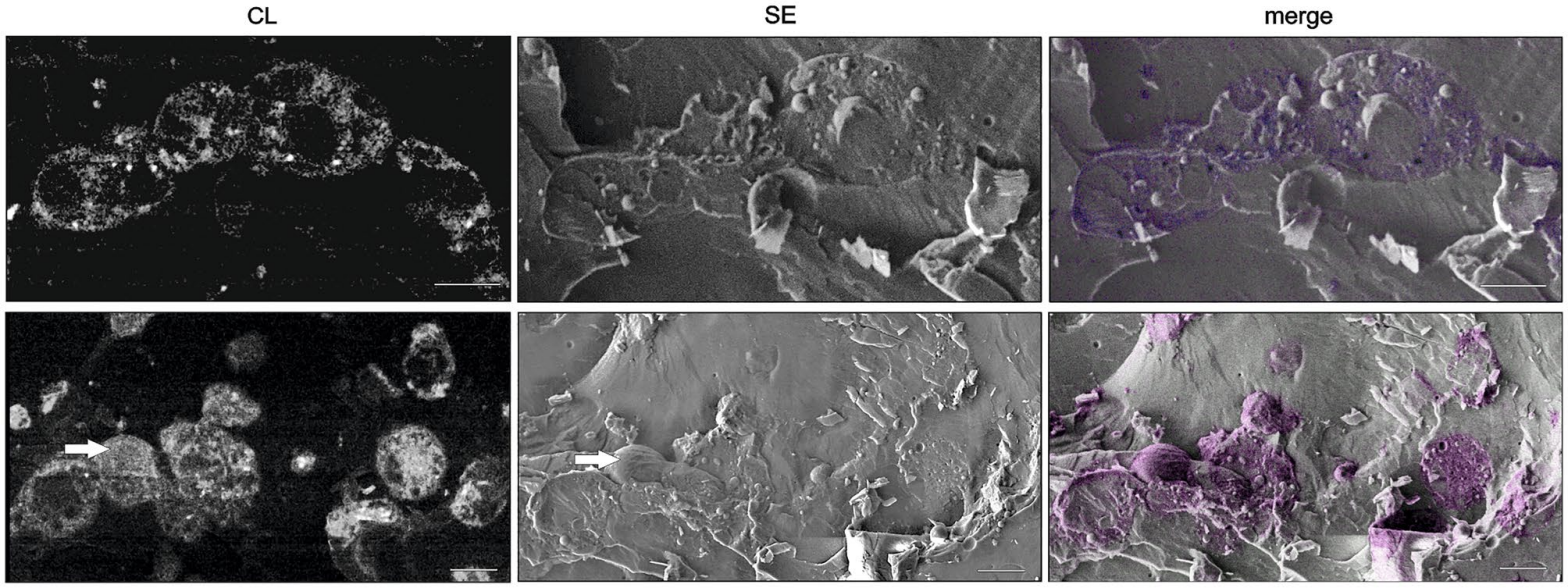
SEM of freeze fractured Vero cells stained in vivo with REZOLVE-ER. Corresponding images were taken using Crytur cathodoluminescence detector (CL) and the Everhart–Thornley detector (SE) at − 140 °C and 5 keV. The CL image was colourised and merged with the SE image to illustrate the exact structural overlay. The CL signal helped find positions of stained cells in the fractured area and identify labeled cellular compartments. The arrow marks the intact cell. SEM JEOL 7401F. Bars: 10 µm.

**Figure 3:**
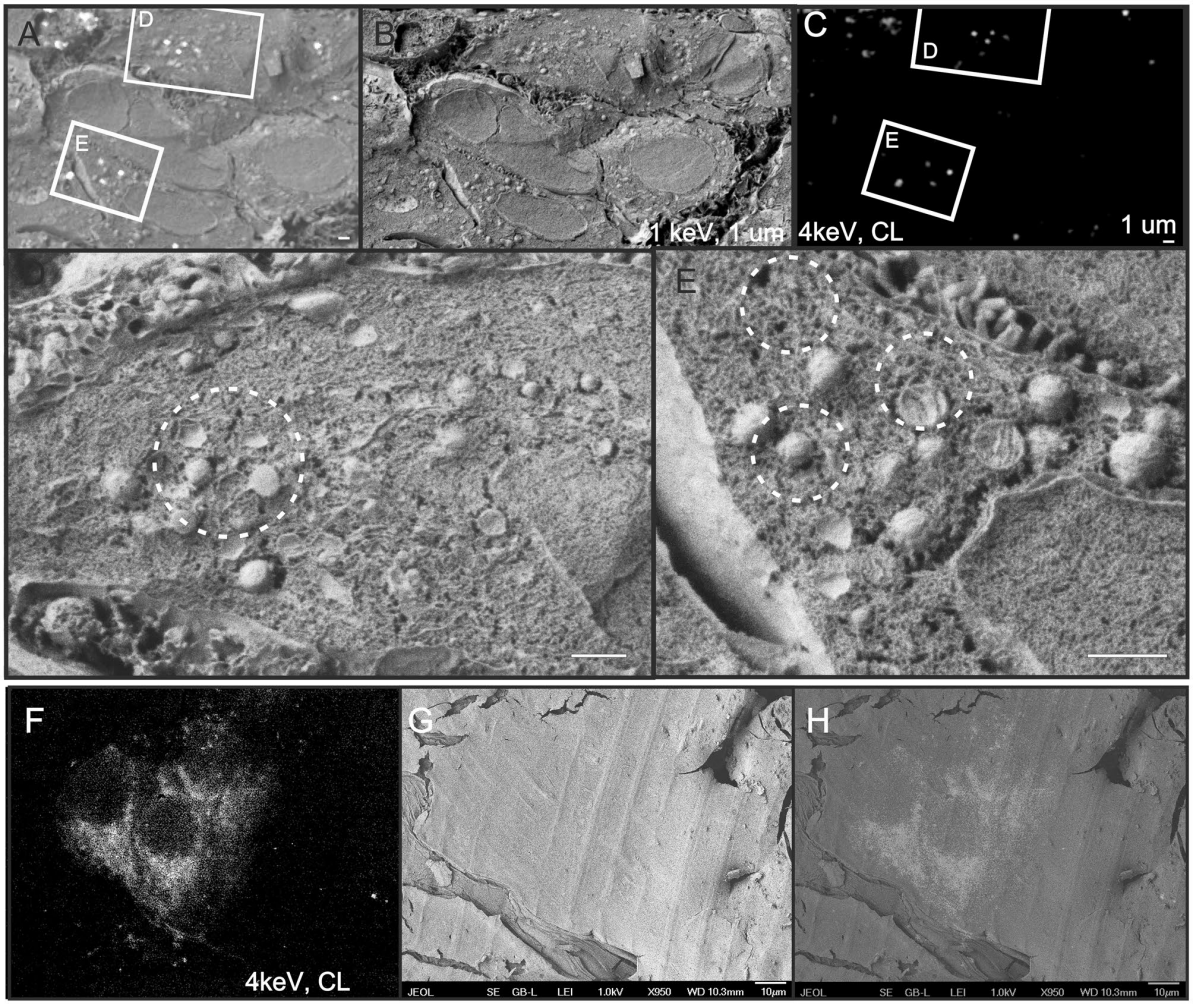
Correlative cryo-cathodoluminescence (CL) scanning electron microscopy of Vero cells stained with IRAZOLVE-MITO. (**A**–**E**) In vivo stained cells were frozen, fractured, and imaged at − 135 °C using Crytur CL detector (4 keV) and the Everhart–Thornley detector (ETD, 1 keV). (**F**–**H**) Chemically pre-fixed cells were stained, frozen at high pressure, and corresponding images were taken at 4 keV using CL (**F**) and ETD (**G**). Bars: 1 µm (**A**–**E**), 10 µm (**F**–**H**). SEM JEOL 7401F. CL and topographic images were merged manually (**A**,**H**).

CL imaging was performed on stained, high-pressure frozen cells that were freeze-fractured before cryo-scanning electron microscopy (cryo-SEM) observation. We found that REZOLVE-ER (Fig. 2, left panel) and IRAZOLVE-MITO (Fig. 3) exhibit strong CL signal that closely coincided with the fluorescence (Fig. 1).

In the cryo-SEM, CL enabled quick mapping of stained organelles over large areas and allowed to distinguish from intact cells embedded within the vitrified medium, which facilitated navigation to the regions of interest. At low magnification, CL clearly revealed the shape of cells and nuclei position. The consecutive secondary electron images provided topographic data of the sample, which allowed for identification of additional organelles (Fig. 2, left panel; Fig. 3D,E). Since the acquisition parameters did not match for the topographic (1 kV) and CL (4 kV) measurements, the registration was performed manually by using defined landmarks. Unstained cells were used as negative controls to confirm that the settings used for visualization of both IRAZOLVE-MITO and REZOLVE-ER did not detect endogenous CL (data not shown).

We also aimed to find how the individual steps of the conventional SEM preparation protocol at room temperature affect the CL signal. In contrast to frozen-hydrated cells (Fig. S1A), aldehyde-fixed cells dehydrated using organic solvents (ethanol, acetone) at room temperature did not display CL signal. The CL signal was partially preserved after air-drying (or rotary-pump drying); however, it bleached almost immediately under the electron beam excitation (Fig. S1B,C).

**Figure 4:**
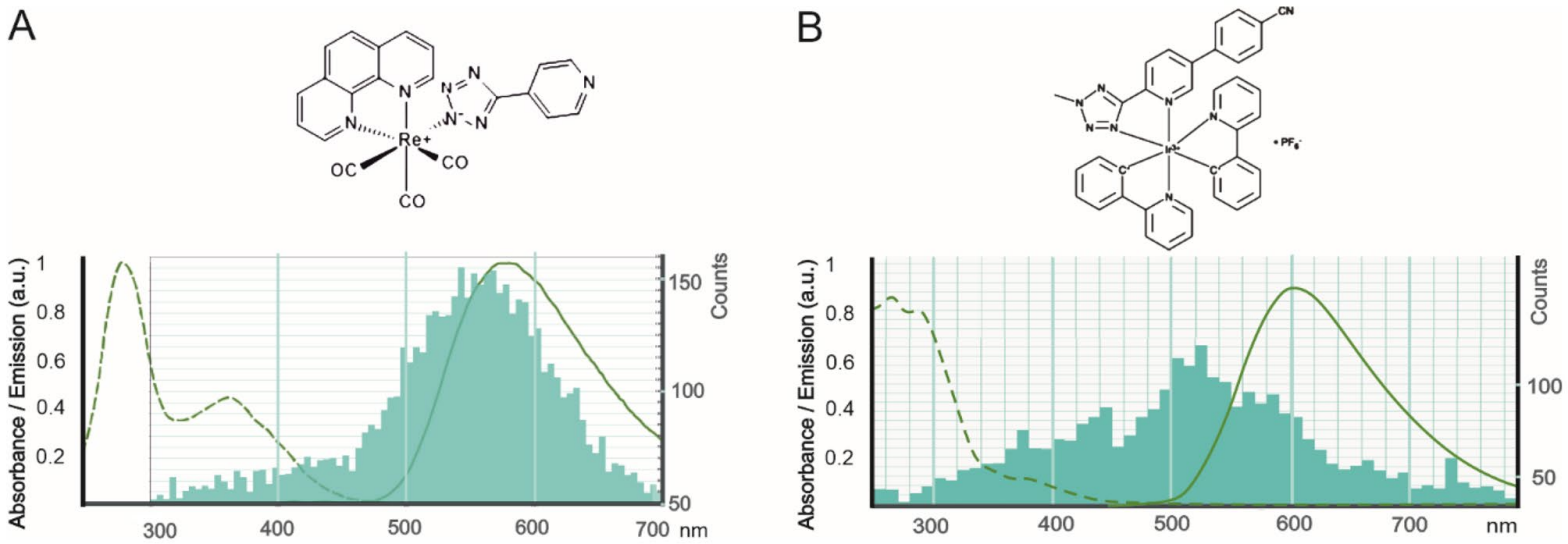
The CL emission spectra of REZOLVE-ER (**A**) and IRAZOLVE-MITO (**B**) probes are shown in turquoise. (**A**) For comparison, the absorbance/emission spectrum of REZOLVE-ER is 350–405 nm/500–650 nm (green) according to the datasheet. The broad emission band of IRAZOLVE-MITO in CH_2_Cl_2_ is centered at ~ 605 nm^24^. CL emission spectra were measured by the MonoCL4 detector at − 135 °C. The dominating were bands at approx. 550 nm for REZOLVE-ER (**A**) and 525 nm for IRAZOLVE-MITO dye (**B**).

The CL was taken independently using two distinct CL detectors: the Crytur CL detector (Figs. 2, 3, Figs. S1, S3, S5) or the MonoCL4 + CL detector from Gatan (only Fig. S2) mounted to the SEM Magellan 400L, where we also measured the CL emission spectra at 5 kV, 0.1 nA, and − 140 °C. The CL spectrum of REZOLVE-ER staining showed a dominant initial emission band centered around 550 nm (Fig. 4A), which corresponded with an emission profile for REZOLVE-ER between 500 and 650 nm^27^. The dominating CL emission band of IRAZOLVE-MITO was in the 520 nm region (Fig. 4B). However, the absorbed electron dose, which linearly increased during serial CL spectral acquisition, could bleach the iridium complexes and thus impair the recording of spectra at longer wavelengths. The IRAZOLVE-MITO excited at 403 nm had an emission interval from 505 to 625 nm^24^. Both CL emission maxima were obtained at -140 °C and are presumably blue-shifted compared to luminescence emission maxima obtained at room temperature. The blue shift of luminescence emission maxima of organometallic complexes obtained at − 196 °C versus room temperatures was observed earlier and explained with the rigidochromic effect^27^.

The lowest accelerating voltage generating the CL emission of both probes was 2 kV; however, the optimal signal-to-noise ratio was obtained at 4–5 kV (Fig. S3A) and 30–300 pA  probe currents (Fig. S3B,C).

At these parameters, we estimated the spatial and lateral resolution of the CL signal based on the Monte Carlo simulation of electron scattering. The simulations allow to quantitatively estimate the CL generation in a bulk sample (here amorphous ice as the predominant part of a biological sample) within the electron interaction volume (Fig. S4A). The highest CL intensity around the actual beam position decreases significantly with increasing radius but still interferes into hundreds of nm with significant background (Fig. S4B). In the z-axis, we estimated that most of the CL signal is generated at depths around 250 nm under the surface. It is given by significantly higher irradiated volume even if less than 20% of initial beam intensity reaches this depth. In *xy*-plane, most of the signal (more than 60%) is generated from a radius < 8 nm (simulated beam diameter is 4 nm). These theoretical values are strongly affected by sample inhomogeneities, CL emitting particles/compounds distribution or light refraction/absorption in real samples. The resolution can be enhanced only with decreasing primary beam energy or using an electron transparent sample where the characteristic electron interaction pear-shape is not being fully developed. Next limiting factors are CL detector sensitivity, working distance, and stability of specimens under the electron beam.

**Figure 5:**
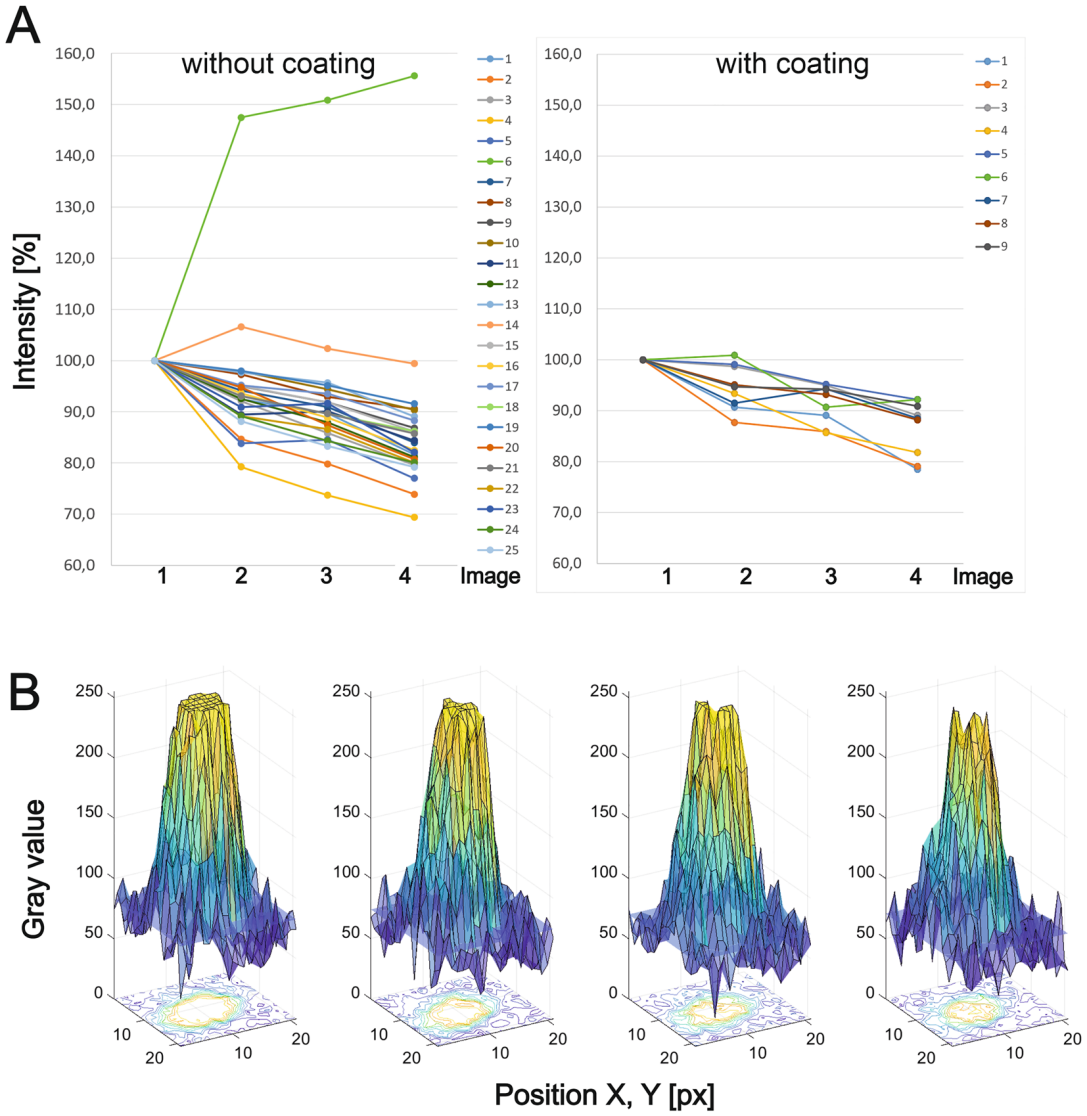
Decay of cathodoluminescence intensities of IRAZOLVE-MITO dye (without and with sputtered gold nanolayer) measured on four consecutive images taken at the same parameters of the CL detector and the SEM (4 keV, 15 µs, 300 pA, 10,367 px/nm). (**A**) The graph shows changes in CL intensities measured at the first and consecutive three scans. (**B**) The CL intensities were measured over the representative uncoated area (area 25 from Fig. S5 A is shown) from first to fourth images (from left to right). The blue plane represents the level of background mean value.

Under selected parameters (4 kV, 15 µs, 300 pA, − 140 °C), we compared the decay of the CL signal from intensity profiles measured on the IRAZOLVE-MITO stained structures without and with gold sputter coating (~ 2 nm) (Fig. 5). From measurements of CL intensities over 25 uncoated CL areas from the first and the following three SEM images, we calculated the mean pixel intensity for each image (area 6 was excluded, see Fig. 5A; Fig. S5).

We found that the CL signal decreased between the acquisition of the first and the second SEM image in uncoated samples by ~ 7.4%, then ~ 3.4% and ~ 5.5% to the value of ~ 83.8% (the fourth SEM image) (Fig. 5A, left). In the coated samples, CL decreased by ~ 5.4%, then ~ 3.2%, and ~ 4.8% to the value of ~ 86.7% (Fig. 5B, right). The gray-scale intensities of one selected area are shown in Fig. 5B. In comparison, organic fluorophores bleached under the electron beam (2 kV, 0.8 nA, equivalent to a dose of 0.04 nC/μm^2^) almost immediately^19^. Quantum dots (CdSe/CdS) exhibited highly reduced CL emission (by 30%) within the first 10 s (equivalent to a dose of 0.08 nC/μm^2^), whereas the drop in emission intensity of rare-earth element-doped nanocrystals was 10–20% within the first 10 s (equivalent to a dose of 0.04 nC/μm^2^)^19^.

Here, we demonstrated that IRAZOLVE-MITO and REZOLVE-ER dyes^21,22^ produce CL signal, which can be used for correlative studies combining live and CL-SEM imaging at cryogenic temperatures. The advantage of this approach is that one can perform localization studies and structural imaging at cryogenic temperatures simply in one SEM instrument without multiple sample transfers typical for non-integrated cryo-fluorescence-SEM workflows^4,5^. Cryo CL-SEM imaging approach may unleash the potential of the CL to localize molecules in the cellular volume using the cryo-FIB-SEM, cryo-block-face imaging techniques, and it can be an alternative to integrated cryo-correlative fluorescence electron microscopy workflow (e.g., “iCorr” or Photon Ion Electron microscope)^28,29^.

In contrast to rare-earth element doped nanocrystals and nanodiamonds mainly used to specifically label components in the endocytic pathway, IRAZOLVE-MITO and REZOLVE-ER have been designed for bioimaging as alternative organelle-specific dyes. Widespread application of CL in bio-imaging is currently limited by the availability of high-intensity CL probes targeting structures of interest, their insufficient stability under the electron beam, and lack of probes with distinct emission spectra that would allow multicolor CL-SEM. CL properties also need to be tested for IRAZOLVE-ALKYNE and REZOLVE-ALKYNE tags as well as other novel photoluminescent iridium or rhenium complexes, now well-established probes used in life science research^23,30–32^. For example, rhenium(i) tricarbonyl complexes incorporating 1,10-phenanthroline or the 3-chloromethylpyridyl bipyridine that accumulate in mitochondria^33,34^, a rhenium complex linked to 4-cyanophenyltetrazolate (commercially available as REZOLVE-L1), which has an affinity to polar lipids (specifically to phosphatidylethanolamine, sphingomyelin, sphingosine), and finally a rhenium 3-pyridyltetrazolate complex, which localizes within the lysosomes^34^. Another new probe, a cyclometalated iridium complex, was designed to visualize microtubules by light and electron microscopy^35^. Phosphorescent organometallic iridium complexes have also been recently used to detect diatoms viability^36^. Other luminescent probes include iridium (III) polypyridine complexes^37^, which exhibit photocytotoxic activity upon prolonged laser irradiation^38^.

In conclusion, this study shows that luminescence probes from the group of iridium and rhenium complexes, IRAZOLVE-MITO and REZOLVE-ER, generate detectable CL signals in vitrified samples at low electron beam energies. We carried out CL and topography imaging on freeze fractured cells and found that the CL intensity of IRAZOLVE-MITO dye was reduced by 16% in uncoated samples and 13% in coated samples between the first and fourth CL image acquisition of the same region taken at − 140 °C, 4 kV, 300 pA, and 15 µs. As CL imaging can be easily integrated into simultaneous multimodal imaging offered by the modern SEM, we believe in great potential of using organelle-specific probes such as IRAZOLVE-MITO and REZOLVE-ER in high-resolution biological imaging. The dyes can be used to select target regions before cryo-FIB milling or perform correlative CL-SEM as demonstrated in this study.

## Methods

### Cultures and staining and in vivo imaging

Human airway epithelial cells A549 were cultivated in DMEM Low glucose medium with 10% bovine fetal serum, 1% ATB (Penicillin, Streptomycin), and 1% Glutamine Stable at 37 °C and 5% CO_2_. Cell cultures of Vero E6 were cultivated in FLUOROBRITE DMEM high glucose medium with ATB at 37 °C and 5% CO_2_. The cells were seeded to 25 cm^2^ tissue culture flasks or µ-Dish 35 mm Imaging Chamber with a glass-bottom (Ibidi) to achieve 80–100% confluence. REZOLVE-ER and IRAZOLVE-MITO (both ReZolve Scientific, see acknowledgments for further details) were dissolved in DMSO to 10 mM (stock solutions). Cells were stained in the presence of culture media by adding either the REZOLVE-ER stock solution (to final concentrations of either 33 or 50 µM) or IRAZOLVE-MITO (20 µM, 40 µM). Cells were imaged in vivo by a holographic microscope 3D Cell Explorer (Nanolive) either after 30 min or overnight incubation. In the case of IRAZOLVE-MITO staining, the cells were stained and imaged after fixation in 4% formaldehyde in 0.1 M HEPES for 1 h at room temperature, followed by a 30 min wash step.

### Cryo-scanning electron microscopy and CL imaging

Pelleted cells were high pressure frozen (EM ICE, Leica Microsystems) in the presence of 20% bovine serum albumin and transferred into the scanning electron microscope (SEM) JEOL 7401F (JEOL Ltd.) equipped with a cryo-attachment CryoALTO 2500 (Gatan, Inc.) precooled to − 140 °C. Cells were freeze- fractured, optionally freeze-etched at temperatures between − 98 °C and − 95 °C for 10 s, and gold-coated for 10 s with gold (~ 2 nm). Images were taken using the Everhart–Thornley detector of secondary electrons and the CL detector designed by Crytur comp. The CL detector detects panchromatic CL signals with a high signal-to-noise ratio in the spectral range 330–600 nm. This retractable CL detector of the parabolic mirror type consists of a quartz light guide and the photomultiplier. The contrast and brightness settings of the CL detector during CL intensity measurements were constant.

Independent observation was performed using the SEM Magellan 400L (ThermoFisher Scient.) equipped with MonoCL4 + CL detector (Gatan, Inc.) at a range of temperatures from − 125 °C to − 150 °C. Freeze fracturing and Au/Pt coating (appr. ~ 2.7 nm) was performed using the sputter coater Leica ACE 600 (Leica Microsystems). CL images and spectra were taken at a beam energy of 7 keV and probe current of 0.1 nA.

### Preparation of cells for observation by SEM at room temperature

Cells stained in vivo by IRAZOLVE-MITO were fixed in 4% formaldehyde and 0.1% glutaraldehyde in 0.1 M HEPES for either 15 min or overnight. After washing, cells were, dehydrated with graded acetone series followed by two washes in 100% acetone. After critical point drying, the glass cover slides were mounted onto stubs and coated for 10 s using gold (Baltec SCD 050). Stained and fixed cells were optionally air-dried or dried with a rotary pump without dehydration.

### Image analysis of intensity and decay of CL

At first, we took four consecutive images at the same parameters (4 keV, 15 µs, 300 pA) of Vero cells stained in vivo with IRAZOLVE-MITO dye without coating or gold-coated (~ 2 nm) using the Crytur CL detector mounted to SEM JEOL 7401F at − 140 °C. Intensities and decay of CL were analyzed from images using the Matlab tool as shown in Fig. S5. The centers with the highest CL intensities from 25 areas from the uncoated specimen and 9 areas from the gold-coated specimen were detected by FAST-PEAK-FIND^39^ (Fig. S5A), with a threshold parameter equal to 145 as determined manually from the maximum intensity of cells in the background. For further analysis, we cut each area with the highest CL intensities from the image out to a square area in size of 21 × 21 px (Fig. S5B). For each area, we determined the diameter of the circle defining the CL region (marked red in Fig. S5B) as the maximum of the second derivative (marked red in Fig. S5D) obtained from the average intensities calculated in concentric circles with an increasing diameter with the centre located in the middle of this square area (Fig. S5B, C). Total CL intensity was calculated and statistically analyzed from this internal CL region (Fig. S5E) whereas the outer areas represented the background (Fig. S5F). CL intensities were plotted over time (four consecutive photos), where the first value represents 100% (Fig. 5).

The CL resolution simulated for amorphous ice and beam energy of 4 keV in a bulk sample was computed in Casino 2.5^40^.

## Supplementary Information

Supplementary Information.

## Data Availability

All data generated or analysed during this study are included in this published article and its supplementary information files.

## Abbreviations

CL: Cathodoluminescence
SEM: Scanning electron microscopy

## References

[1] Tabata S (2019). Electron microscopic detection of single membrane proteins by a specific chemical labeling. iScience.

[2] Bakkum T (2020). Bioorthogonal correlative light-electron microscopy of mycobacterium tuberculosis in macrophages reveals the effect of antituberculosis drugs on subcellular bacterial distribution. ACS Cent. Sci..

[3] Kaufmann R (2014). Super-resolution microscopy using standard fluorescent proteins in intact cells under cryo-conditions. Nano Lett..

[4] Strnad M (2015). Correlative cryo-fluorescence and cryo-scanning electron microscopy as a straightforward tool to study host-pathogen interactions. Sci. Rep..

[5] Vancová M (2017). Pleomorphism and viability of the lyme disease pathogen Borrelia burgdorferi exposed to physiological stress conditions: A correlative cryo-fluorescence and cryo-scanning electron microscopy study. Front. Microbiol..

[6] Pease RFW, Hayes TL (1966). Scanning electron microscopy of biological material. Nature.

[7] Zielinski MS (2019). Quantitative intrinsic auto-cathodoluminescence can resolve spectral signatures of tissue-isolated collagen extracellular matrix. Commun. Biol..

[8] De Mets M, Lagasse A (1971). An investigation of some organic chemicals as cathodoluminescent dyes using the scanning electron microscope. J. Microsc..

[9] Fisher PJ, Wessels WS, Dietz AB, Prendergast FG (2008). Enhanced biological cathodoluminescence. Opt. Commun..

[10] Akiba K, Tamehiro K, Matsui K, Ikegami H, Minoda H (2020). Cathodoluminescence of green fluorescent protein exhibits the redshifted spectrum and the robustness. Sci. Rep..

[11] Fern GR, Silver J, Coe-Sullivan S (2015). Cathodoluminescence and electron microscopy of red quantum dots used for display applications. J. Soc. Inform. Display.

[12] Keevend K (2017). Tb3+-doped LaF3 nanocrystals for correlative cathodoluminescence electron microscopy imaging with nanometric resolution in focused ion beam-sectioned biological samples. Nanoscale.

[13] Nagarajan S (2016). Simultaneous cathodoluminescence and electron microscopy cytometry of cellular vesicles labeled with fluorescent nanodiamonds. Nanoscale.

[14] Prigozhin MB (2019). Bright sub-20-nm cathodoluminescent nanoprobes for electron microscopy. Nat. Nanotechnol..

[15] Keevend K (2019). Ultra-bright and stable luminescent labels for correlative cathodoluminescence electron microscopy (CCLEM) bioimaging. Nano Lett..

[16] Glenn DR (2012). Correlative light and electron microscopy using cathodoluminescence from nanoparticles with distinguishable colours. Sci. Rep..

[17] Keevend K, Coenen T, Herrmann IK (2020). Correlative cathodoluminescence electron microscopy bioimaging: Towards single protein labelling with ultrastructural context. Nanoscale.

[18] Fukushima S (2016). Correlative near-infrared light and cathodoluminescence microscopy using Y2O3:Ln, Yb (Ln = Tm, Er) nanophosphors for multiscale, multicolour bioimaging. Sci. Rep..

[19] Keevend K (2019). Ultrabright and stable luminescent labels for correlative cathodoluminescence electron microscopy bioimaging. Nano Lett..

[20] Furukawa T (2015). Rare-earth-doped nanophosphors for multicolor cathodoluminescence nanobioimaging using scanning transmission electron microscopy. J. Biomed. Opt..

[21] Bader CA (2016). Imaging nuclear, endoplasmic reticulum and plasma membrane events in real time. FEBS Lett..

[22] Sorvina A (2018). Mitochondrial imaging in live or fixed tissues using a luminescent iridium complex. Sci. Rep..

[23] Caporale C, Massi M (2018). Cyclometalated iridium(III) complexes for life science. Coord. Chem. Rev..

[24] Caporale C (2017). Investigating intracellular localisation and cytotoxicity trends for neutral and cationic iridium tetrazolato complexes in live cells. Chemistry.

[25] Bader CA (2014). Modulation of the organelle specificity in Re(i) tetrazolato complexes leads to labeling of lipid droplets. RSC Adv..

[26] Sánchez MI (2020). MitoBlue as a tool to analyze the mitochondria-lysosome communication. Sci. Rep..

[27] Wright PJ (2012). Synthesis, photophysical and electrochemical investigation of dinuclear tetrazolato-bridged rhenium complexes. Organometallics.

[28] Wang S, Li S, Ji G, Huang X, Sun F (2017). Using integrated correlative cryo-light and electron microscopy to directly observe syntaphilin-immobilized neuronal mitochondria in situ. Biophys. Rep..

[29] Gorelick S (2019). PIE-scope, integrated cryo-correlative light and FIB/SEM microscopy. eLife.

[30] Liu Z, Bian Z, Huang C, Hubert B, Véronique G (2010). Molecular Organometallic Materials for Optics.

[31] Dixon IM (2000). A family of luminescent coordination compounds: Iridium() polyimine complexes. Chem. Soc. Rev..

[32] Caporale C (2020). Photophysical and biological properties of iridium tetrazolato complexes functionalised with fatty acid chains. Inorganics.

[33] Amoroso AJ (2008). 3-Chloromethylpyridyl bipyridine fac-tricarbonyl rhenium: A thiol-reactive luminophore for fluorescence microscopy accumulates in mitochondria. New J. Chem..

[34] Bader C (2014). Modulation of the organelle specificity in Re( I) tetrazolato complexes leads to labeling of lipid droplets dagger. RSC Adv..

[35] Tian X (2020). A cyclometalated iridium (III) complex as a microtubule probe for correlative super-resolution fluorescence and electron microscopy. Adv. Mater..

[36] Leone G (2020). A phosphorescent iridium complex as a probe for diatom cells’ viability. MRS Adv..

[37] Lo KK-W (2015). Luminescent rhenium(I) and iridium(III) polypyridine complexes as biological probes, imaging reagents, and photocytotoxic agents. Acc. Chem. Res..

[38] Yip A, Lo KK-W (2018). Luminescent rhenium(I), ruthenium(II), and iridium(III) polypyridine complexes containing a poly(ethylene glycol) pendant or bioorthogonal reaction group as biological probes and photocytotoxic agents. Coord. Chem. Rev..

[39] Natan. *“Fast 2D Peak Finder.” Matlab Central File Exchange*. https://es.mathworks.com/matlabcentral/fileexchange/37388-fast-2d-peak-finder?s_tid=FX_rc1_behav (accessed on 8 July 2021). (2021).

[40] Drouin D (2007). CASINO V2.42: A fast and easy-to-use modeling tool for scanning electron microscopy and microanalysis users. Scanning.

