# Supplementary material for: Cathodoluminescence imaging of cellular structures labeled with luminescent iridium or rhenium complexes at cryogenic temperatures
Source: Sci Rep. 2022 Aug 4;12:13432. doi: 10.1038/s41598-022-17723-w (PMC9352783; doi:10.1038/s41598-022-17723-w)
Supplement: Supplementary file 1 — Supplementary Information. [file 41598_2022_17723_MOESM1_ESM.pdf]

Supporting Information for:

Cathodoluminescence imaging of cellular structures labeled with luminescent iridium or rhenium complexes at cryogenic temperatures

Marie Vancová[1,2,*], Radim Skoupý[3], Eva Ďurinová[1,2], Tomáš Bílý[1,2], Jana Nebesářová[1,4], Vladislav Krzyžánek[3], Aleš Kolouch[5], Petr Horodyský[5]

[1]Institute of Parasitology, Biology Centre CAS, České Budějovice, 37005, Czech Republic

[2]Faculty of Science, University of South Bohemia, České Budějovice, 37005, Czech Republic

[3]Institute of Scientific Instruments CAS, Brno, 612 000, Czech Republic

[4]Faculty of Science, Charles University in Prague, Prague, 128 00, Czech Republic

[5]CRYTUR, spol. s r.o., Turnov, 511 01, Czech Republic

[*]vancova@paru.cas.cz

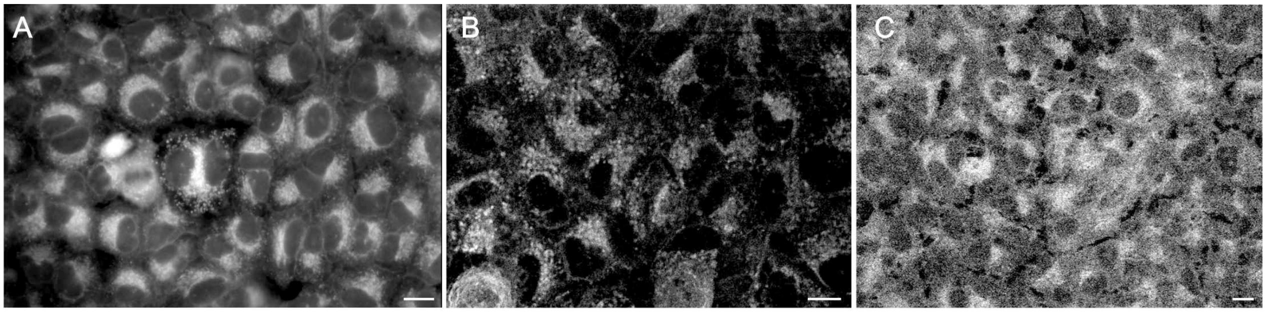

Figure S1. Mitochondria live-stained with IRAZOLVE-MITO visualized in intact whole human airway epithelial cells A549 adhered to glass cover slides and fixed in 4% formaldehyde. A. Fluorescence microscopy. B-C. SEM-CL image. Before SEM observation with JEOL 7401F equipped with the Crytur CL detector, cell monolayers were either HPF-frozen, sublimated for 1 min at -95°C (B), or chemically fixed and air-dried using a rotary pump (C). The CL signal from air dried cells was quickly destroyed in contrast to observation at –135°C. Olympus BX41 (A), 5 keV (B), 4 keV (C). Bars 10 μm.

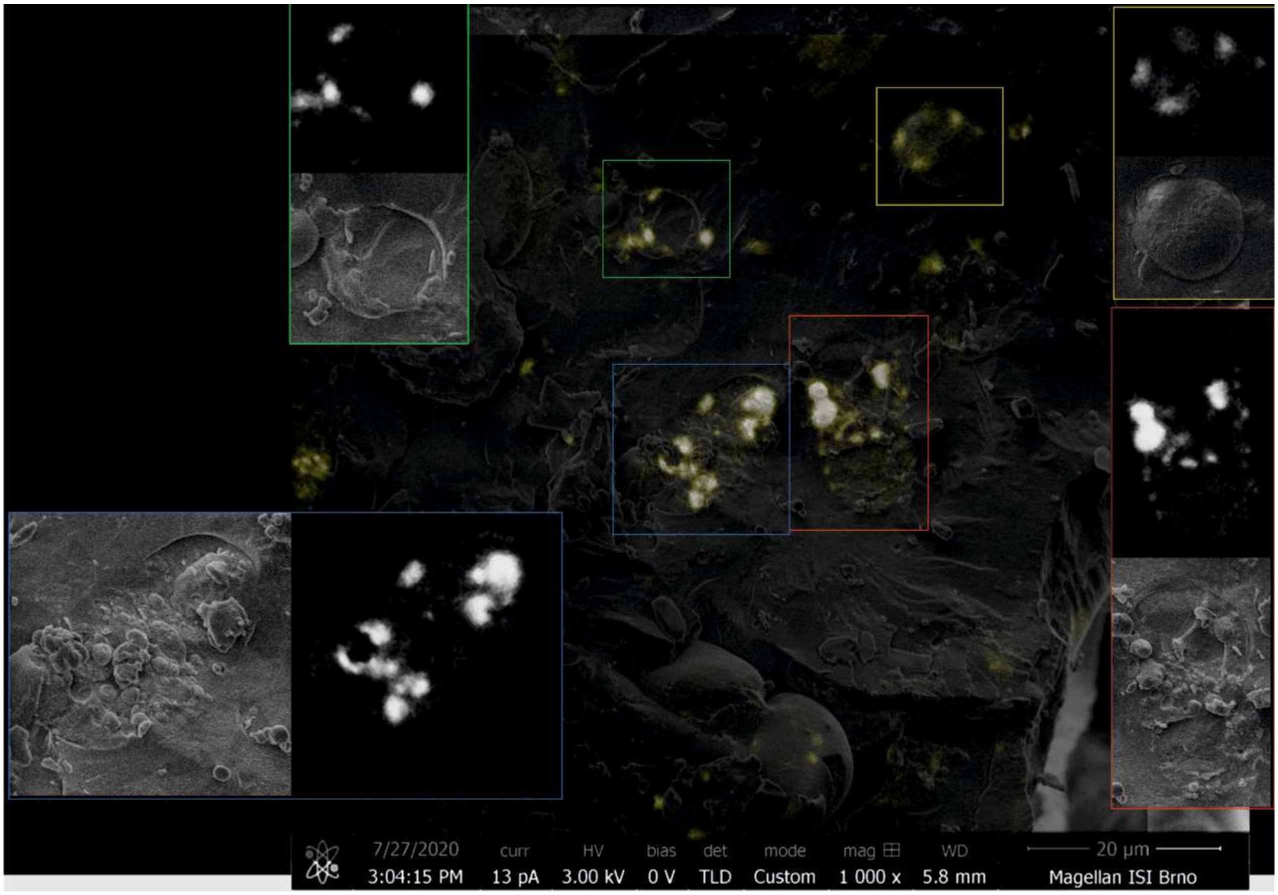

Figure S2. Live-stained VERO cells with IRAZOLVE-MITO were high pressure frozen, freeze-fractures and coated with Au/Pd 3 nm and imaged by MonoCL4+ CL detector from Gatan mounted to SEMs Magellan 400 at 5 keV, 0.1 nA, and at –140°C.

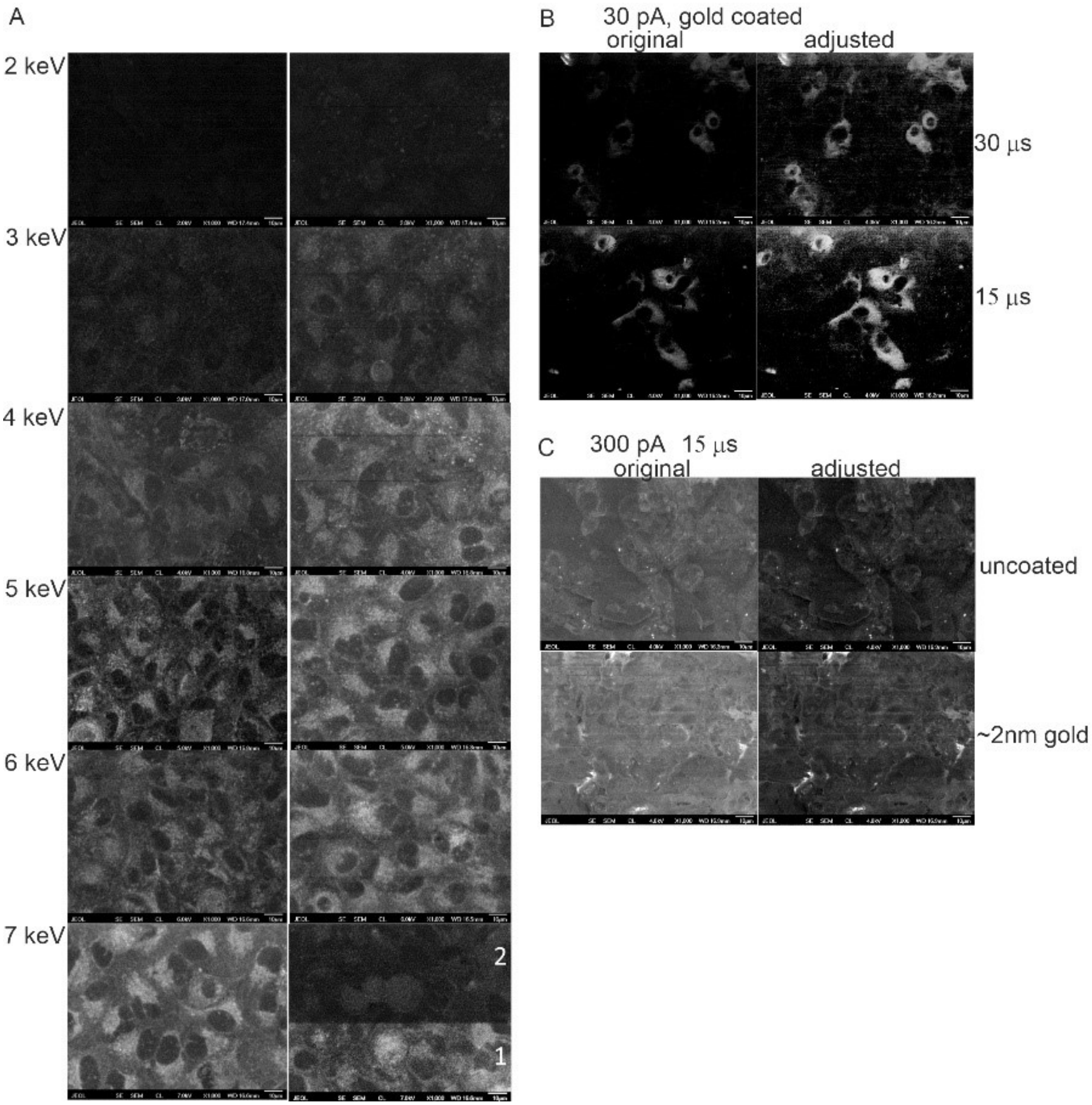

Figure S3. The monolayer of human airway epithelial cells stained with IRAZOLVE-MITO was imaged by the Crytur cathodoluminescence (CL) detector. Cells were high pressure frozen, either uncoated or coated with gold (~ 2nm) and viewed by SEM JEOL 7401F at –135°C. Each image was taken with the CL detector at a new location. A: 2–7 keV, emission current 10 μA, beam current 200 pA, 60 μs dwell time. Working distance from 17.4–16.5 mm. CL detector settings: contrast 94%, brightness 84%. The difference in the intensity of the CL signal at 7 keV after the first and second image captured from the same place. B: CL images recorded at 4 keV and 30 pA. C: CL images were taken at 300 pA current and 15 μs dwell time.

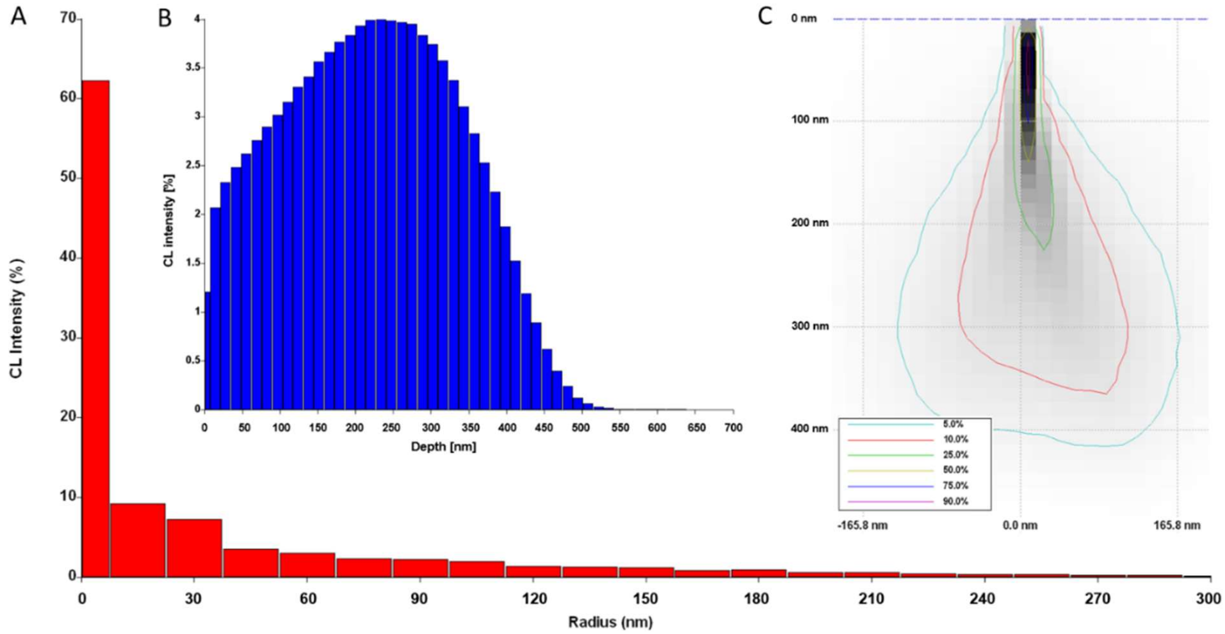

Figure S4. The CL resolution simulated for amorphous ice and beam energy of 4 keV in a bulk sample computed in Casino 2.5. A: Cathodoluminescence intensity distribution according to the radius from the area of electron impact. B: CL intensity distribution by depth. C: Energy distribution in interaction volume.

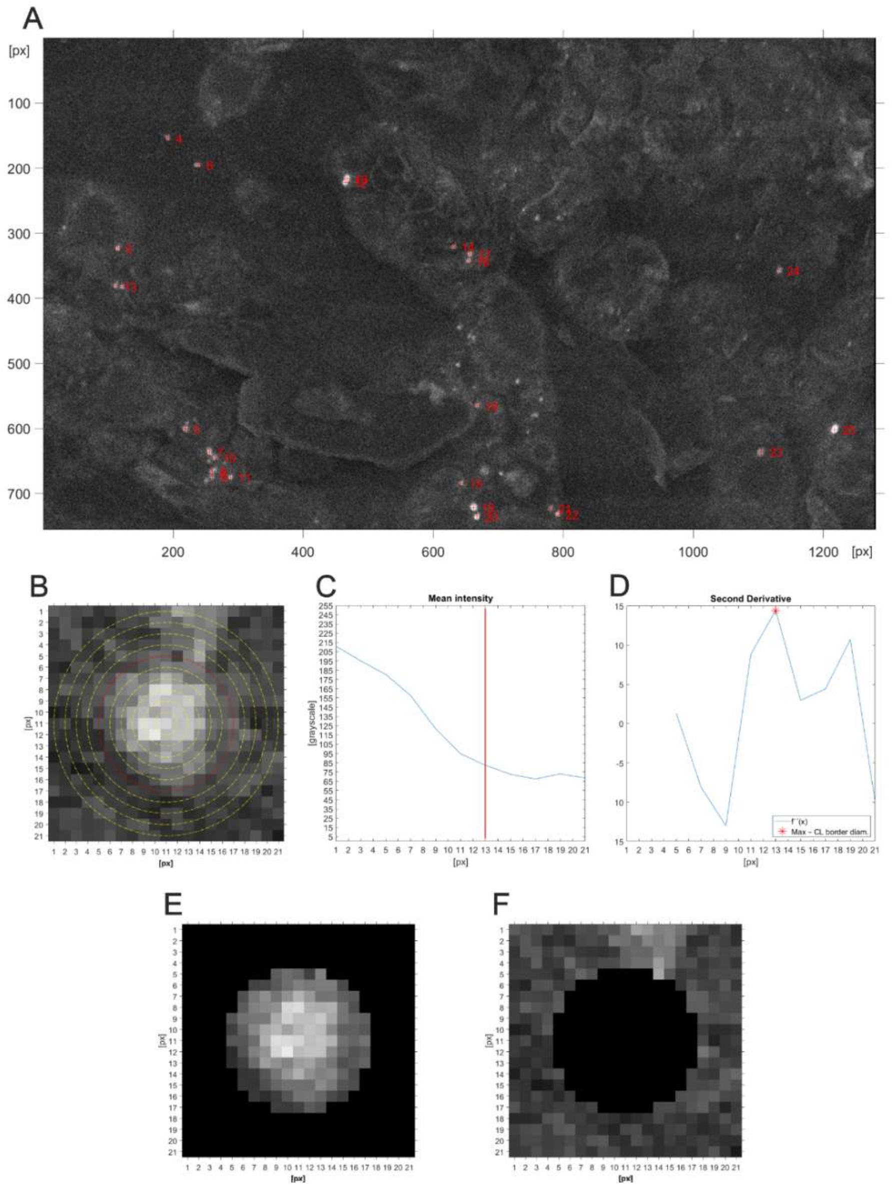

Figure S5. CL intensities were measured and compared using Matlab from four consecutive images of IRAZOLVE-MITO stained Vero cells without coating imaged by cryo-SEM JEOL 7401F. A: Red marked 25 highest CL intensities in the image were detected using FAST-PEAK-FIND. B: A square region 21×21 px with centre specified in step A was cut from the image out for further analysis. C: Mean intensities in concentric circles with an increasing diameter with the centre in the middle of this square area were calculated and plotted into the graph. D: Finding the CL area border as circle diameter is based on finding a maximum of the second derivative of mean intensities (marked red in D also in B and C, respectively). The total intensity was calculated only for CL intensities (E) together with statistics (minimum, mean, standard deviation and maximum) also calculated for background (F). Black pixels complementing the square image were not included in the calculation.
